# Preventive Effect of Vitamin C on Dextran Sulfate Sodium (DSS)-Induced Colitis via the Regulation of IL-22 and IL-6 Production in Gulo(−/−) Mice

**DOI:** 10.3390/ijms231810612

**Published:** 2022-09-13

**Authors:** Hyejung Jo, Dahae Lee, Cheolhyeon Go, Yoojin Jang, Naghyung Chu, Suhyun Bae, Dongmin Kang, Jong Pil Im, Yejin Kim, Jae Seung Kang

**Affiliations:** 1 Laboratory of Vitamin C and Antioxidant Immunology, Department of Anatomy and Cell Biology, Seoul National University College of Medicine, Seoul 03080, Korea; 2 Department of Biology, College of Arts and Sciences, Emory University, Atlanta, GA 30322, USA; 3 Department of Psychological and Brain Sciences, College of Arts and Sciences, Boston University, Boston, MA 02215, USA; 4 Department of Internal Medicine and Liver Research Institute, Seoul National University College of Medicine, Seoul 03080, Korea; 5 Medical Research Center, Institute of Allergy and Clinical Immunology, Seoul National University, Seoul 03080, Korea; 6 Artificial Intelligence Institute, Seoul National University, Seoul 08826, Korea

**Keywords:** vitamin C, IL-22, IL-6, IBD, Gulo(−/−)

## Abstract

Reactive oxygen species (ROS), which are exceptionally high in IBD lesions, are known to cause abnormal immune responses to inflammatory reactions in inflammatory bowel diseases (IBD) through damage to the intestinal mucosal linings. Moreover, they are theorized to be an agent of IBD development. Vitamin C is widely known to be an effective antioxidant for its ability to regulate inflammatory responses through its ROS scavenging effect. Therefore, we examined vitamin C’s influence on the development and progression of IBD in Gulo(−/−) mice, which cannot synthesize vitamin C like humans due to a defect in the expression of L-gulono-γ–lactone oxidase, an essential enzyme for vitamin C production. First, we found extensive oxidative stress and an inflammation increase in the colon of vitamin C-insufficient Gulo(−/−) mice. We also found decreased IL-22 production and NKp46(+) cell recruitment and the impaired activation of the p38MAPK pathway. Additionally, comparing vitamin C-insufficient Gulo(−/−) mice to vitamin C-sufficient Gulo(−/−) mice and wild-type mice, the insufficient group faced a decrease in mucin-1 expression, accompanied by an increase in IL-6 production, followed by the activation of the STAT3 and Akt pathways. The results suggest that vitamin C insufficiency induces severe colitis, meaning vitamin C could also take on a preventative role by regulating the production of cytokines and the induction of inflammation.

## 1. Introduction

Inflammatory bowel disease (IBD), often responsible for abdominal pain, diarrhea, and weight loss, frequently occurs in young adults. Hallmark diseases involving IBD are ulcerative colitis (UC) and Crohn’s disease (CD). Though recognized as a representative intractable inflammatory disease, effective therapeutic agents for IBD have yet to be developed. Therefore, without a definite cure, IBD patients endure a low quality of life. [1]. IBD is generally known to show a high frequency of occurrence in Western countries, but recently, it has been increasing in Asian countries, including Korea, due to changes in the environment and Westernizing diets [2,3,4,5].

Significant advances in our research on intestinal inflammation and its relationship with IBD have shown that it develops through exaggerated immune responses directed toward commensal microbiota [6,7,8]. However, the etiology of IBD ultimately remains inconclusive. Despite some uncertainties, it appears that the depletion of antioxidants coupled with oxidative stress has a substantial role in the development and progression of IBDs [9,10]. Further, it is known that the increased production of free radicals and defects in the antioxidant system needed to remove them are directly related to intestinal damage accompanied by IBD [11,12,13,14,15]. Reactive oxygen species (ROS), a byproduct of oxidative metabolism, are reactive molecules when accompanied by high electronegativity [9]. Although there are some confounding results, patients with IBD generally have more oxidized molecules in different organ systems, namely, the gastrointestinal and respiratory tracts, than healthy controls [16,17,18,19], but the total antioxidant capacity (TAC) and corrected TAC (cTAC) of plasma or serum in patients with UC and CD are, remarkably, lower. Reduced levels of cTAC indicate impaired exogenous antioxidants in IBDs [20]. Among various studies measuring the expression of antioxidants in an eclectic of organs, including colonic tissues, an imbalance in the antioxidant concentration is constant. This supports the idea that IBD patients’ vital organs withstand states of oxidative stress [9].

Vitamin C (L-ascorbic acid) not only donates electrons to enzymatic and nonenzymatic reactions as a reducing agent but is also a robust water-soluble antioxidant. It also functions as an enzymatic cofactor, such as in the synthesis of collagen. Because vitamin C is not endogenously synthesized in humans, it is considered an essential nutrient that must be supplemented through diet. The presence of vitamin C has the benefits of scavenging free radicals, such as antioxidants and anti-inflammatory effects. Conversely, vitamin C deficiency leads to scurvy in humans [21,22,23]. All animals, except humans and some primates, are known to synthesize large amounts of vitamin C in vivo because they express the *gulo* gene, which encodes L-gulono-γ–lactone oxidase (GULO), an essential enzyme for vitamin C biosynthesis. Hence, to determine the effect of vitamin C deficiency on the occurrence and progression of IBD, we conducted our study using animal models that cannot biosynthesize vitamin C to represent humans. Using Gulo(−/−) mice, which mirror human functions regarding vitamin C, we observed the development and progression of IBD due to vitamin C deficiency after inducing acute colitis using dextran sulfate sodium (DSS).

## 2. Results

### 2.1. Vitamin C Insufficiency Increases the Severity of DSS-Induced Colitis and Mortality

**Figure 1:**
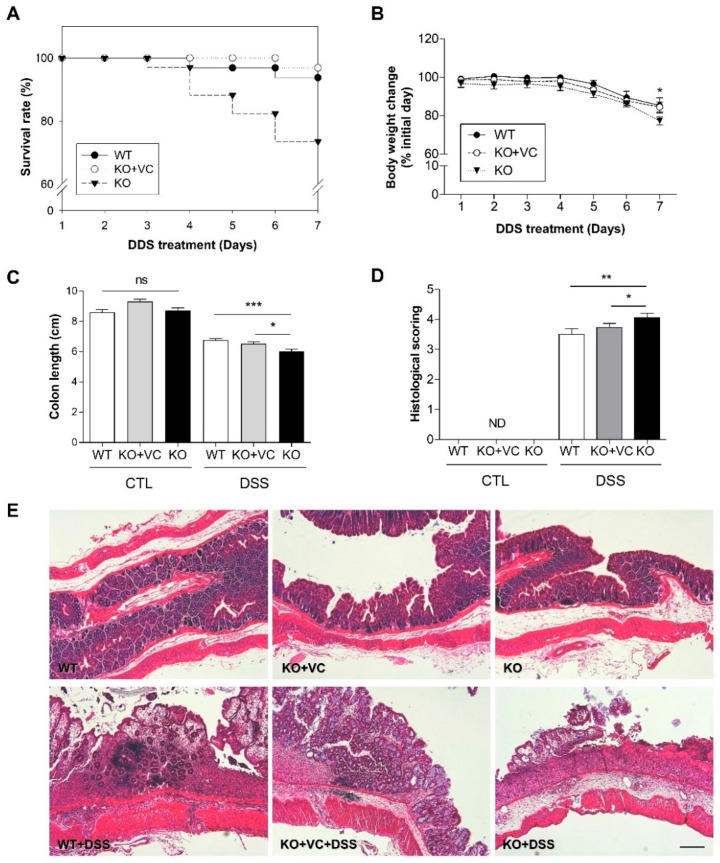
Severe colitis and mortality associated with vitamin C insufficiency and DSS treatment. (**A**) The survival rate and (**B**) body weight of wild-type mice (WT, *n* = 32), vitamin C-sufficient KO mice (KO + VC, *n* = 32), and vitamin C-insufficient KO mice (KO, *n* = 34) were traced for 7 days with 3% DSS treatment. * *p* < 0.05 (WT vs. KO). (**C**) After DSS treatment for 7 days, colon length was measured in each group (*n* = 20–24). ns = not significant, * *p* < 0.05, *** *p* < 0.001. (**D**) Cross-sections of the colon were stained with H&E, and severity was scored according to the criteria described in *Materials and Methods* (*n* = 10). ND = not determined, * *p* < 0.05, ** *p* < 0.01. (**E**) Colons were longitudinally sectioned and stained with H&E. Scale bar, 200 μm. Statistical significance was determined using the Tukey multiple comparisons test after one-way ANOVA.

Wild-type (WT), vitamin C-insufficient Gulo(−/−) (KO), and vitamin C-sufficient Gulo(−/−) (KO + VC) mice were induced with colitis by supplementing drinking water with 3% DSS via drinking for seven days. There were no mortalities among the mice without DSS treatment. However, among the WT and KO + VC groups treated with DSS, the mortality rate was 3–6%, while it was 26% in the KO group (Figure 1A). All mice underwent a decrease in body weight (Figure 1B) and length between the colon and anus to the ileocecal valve in response to DSS; the KO group was affected more dramatically (Figure 1C, Supplementary Figure S1). The severity of colitis was evaluated using a blinded histological scoring system. The histological grade indicated that inflammation was more severe in the KO group than in the control (Figure 1D,E); it revealed that, after DSS treatment, the mice’s colons sustained destruction in the intestinal epithelium with a loss of crypts and epithelial integrity. Additionally, submucosal edema and intense infiltration of inflammatory cells occurred in all layers, which did not occur as gravely for the control group. Taken together, it suggests that vitamin C insufficiency can cause severe colitis and may increase mortality.

### 2.2. Vitamin C Insufficiency Increases Inflammation and Oxidative Stress after DSS Treatment

**Figure 2:**
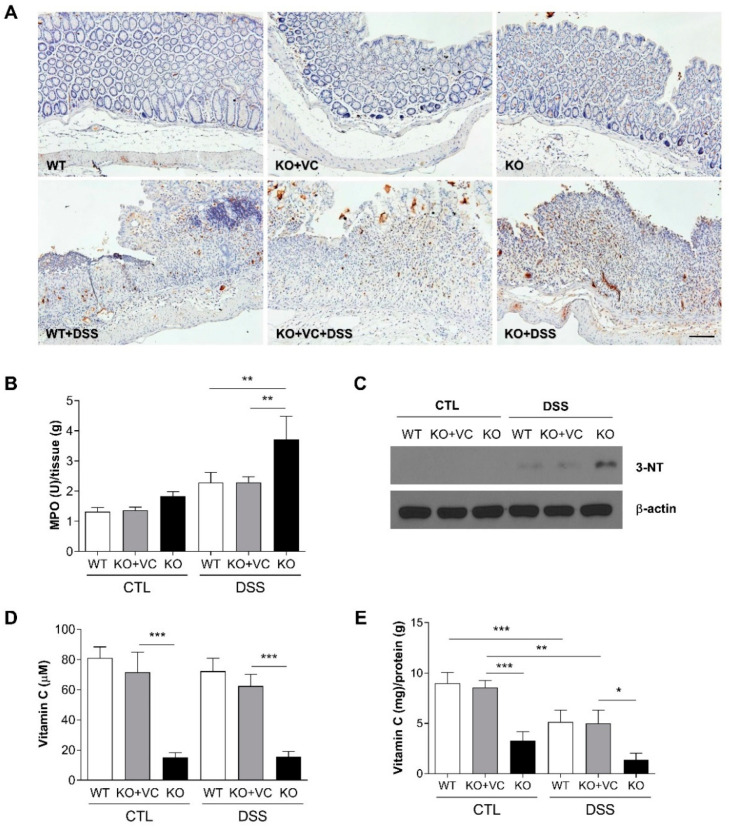
Vitamin C insufficiency and DSS treatment increase inflammation and oxidative stress. WT and KO mice were treated with DSS for 7 days. (**A**) Colons were stained with anti-Gr1. Scale bar, 100 μm. (**B**) The level of MPO was measured in homogenized colon lysates and normalized to tissue weight. (*n* = 15–17). ** *p* < 0.01. (**C**) The expression of 3-NT was evaluated with immunoblotting. The results are representative of three independent experiments. The concentration of vitamin C in (**D**) plasma (*n* = 8–12) and (**E**) colon lysates (*n* = 6–8) was measured using an ascorbate assay kit and normalized to colon weight. * *p* < 0.05, ** *p* < 0.01, *** *p* < 0.001. Statistical analysis was performed using the Tukey multiple comparisons test after one-way ANOVA.

In addition to the histological assessment, we examined the infiltration of immune cells. Gr1-positive cells (mainly neutrophils) infiltrated the colonic epithelium and lamina propria of the DSS-treated groups, especially the DSS-treated KO group (Figure 2A). The activity of myeloperoxidase (MPO), which is abundantly expressed in neutrophils, was significantly increased in the colons of DSS-treated KO mice (Figure 2B). Beyond inflammation, we examined the effect of vitamin C insufficiency on tissues via oxidative stress. Nitrotyrosine (NT), known to increase in various inflammatory diseases such as arteriosclerosis and rheumatoid arthritis, is a marker that indicates the degree of cell damage caused by nitrogen oxide (NO) [24]. By immunoblotting to detect 3-NT in colonic tissues, we found that 3-NT was present in DSS-treated mice and increased in DSS-treated KO mice (Figure 2C). Although the control and DSS-treated groups had almost identical blood levels, the concentration of vitamin C in the blood was 60–80 μM in WT and KO + VC mice and 15–20 μM in KO mice (Figure 2D). DSS treatment decreased the concentration of vitamin C in colonic tissues, and vitamin C levels in colonic tissues were significantly lower in KO mice than in KO + VC mice (Figure 2E). These results suggest that DSS-induced cell infiltration and oxidative stress increased in vitamin C-insufficient KO mice, which might increase the severity of colitis and result in mortality.

### 2.3. DSS-Treated KO Mice with Vitamin C Insufficiency Show Decreased IL-22 Production and p38 MAPK Activation

**Figure 3:**
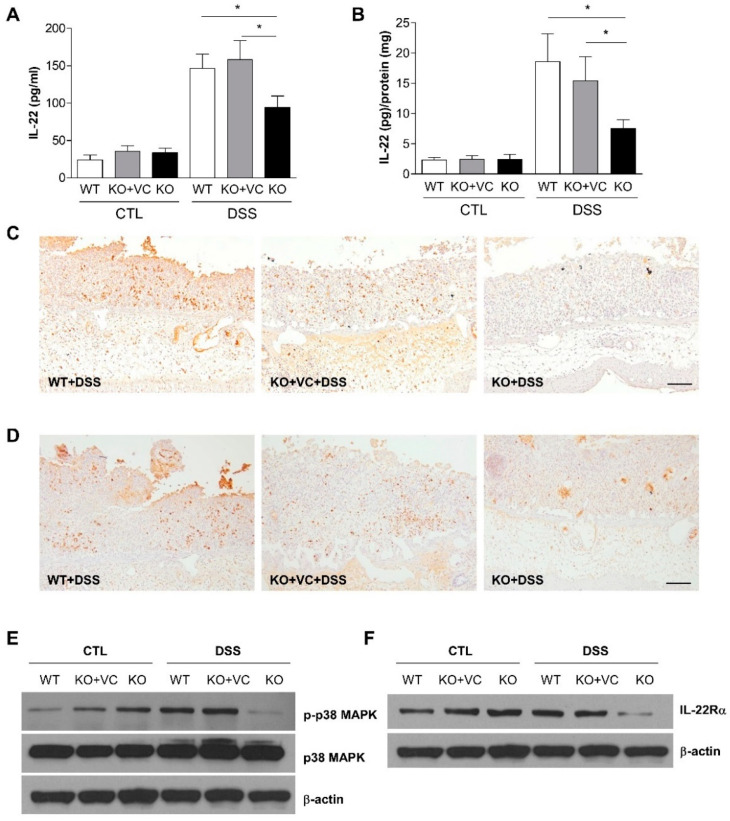
Vitamin C insufficiency and DSS treatment decrease IL-22 production and IL-22Rα expression. Mice were treated with DSS for 7 days, and the level of IL-22 in (**A**) plasma (*n* = 7–11) and (**B**) colon homogenates (*n* = 12–14) was measured with ELISA. The final tissue IL-22 concentration was normalized to proteins in colonic homogenates. * *p* < 0.05. (**C**) IL-22-producing cells and (**D**) the population of infiltrated NKp46(+) cells in the colon were stained using immunohistochemistry. Scale bar, 100 μm. (**E**) The phosphorylation of p38 MAPK in colon lysates was examined with immunoblotting. The results are representative of three independent experiments. (**F**) The expression of IL-22Rα was examined by immunoblotting. β-actin was used as an internal control. The results are representative of three independent experiments. Statistical analysis was performed using the Tukey multiple comparisons test after one-way ANOVA.

**Figure 4:**
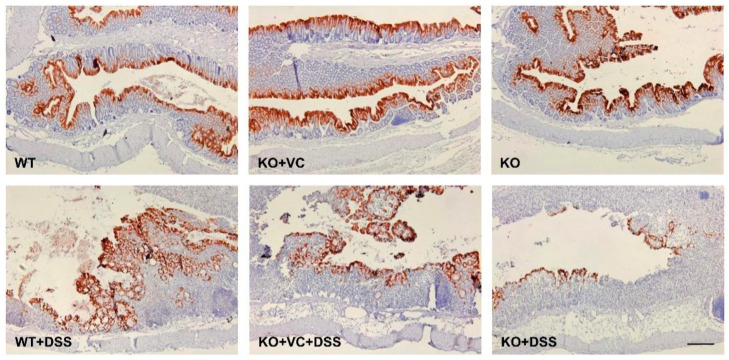
Vitamin C insufficiency and DSS treatment decrease the expression of mucin-1. Mice were treated with DSS for 7 days, and mucin-1 expression was examined by immunohistochemistry. Scale bar, 200 μm.

IL-22, belonging to the IL-10 family of cytokines, targets non-hematopoietic cells such as intestinal epithelial cells, keratinocytes, and hepatocytes because the IL-22 receptor (IL-22R) is expressed only in non-hematopoietic cells and not in hematopoietic cells [25,26]. IL-22 has both pro-inflammatory and anti-inflammatory roles in tissues depending on the inflammatory context. Previous work from our group using the mouse Th1/Th2/Th17 FlowCytomix Multiplex kit resulted in decreased plasma levels of IL-10 in DSS-treated KO mice; therefore, we examined the production of IL-22 by employing the enzyme-linked immunosorbent assay (ELISA) and immunohistochemistry. As shown in Figure 3A, the plasma concentration of IL-22 increased upon DSS treatment, although the increase was less pronounced in the KO group. IL-22 production in colonic tissues was lower in KO mice than in WT and KO + VC mice after DSS treatment (Figure 3B,C). It is known that NKp46, an activating receptor of NK cells, expressing natural killer (NK) cells produces IL-22 in mice and may be involved in intestinal homeostasis and immune defense [27]. An immunohistochemical analysis of the population of NKp46(+) cells in the colon showed that DSS treatment induced a considerable accumulation of NKp46(+) cells. However, the colons of KO mice had a smaller NKp46(+) cell population (Figure 3D) consistent with the levels of IL-22 in Figure 3B,C. Because IL-22 activates STAT3, ERK1/2, JNK, and p38 MAPK downstream of its specific receptor, IL-22Rα, we examined p38 MAPK phosphorylation (Figure 3E) and found that p38 MAPK activation was lower in DSS-treated KO mice, which could be attributed to the lower production of IL-22 in this group. We also examined the expression of IL-22Rα in DSS-treated KO mice because heterodimeric receptors consisting of IL-22Rα and IL-10Rβ recognize IL-22 [28,29]. The results showed that IL-22Rα expression was lower in DSS-treated KO mice (Figure 3F). Based on our findings, in which IL-22 contributes to the rapid attenuation of inflammation in a mouse model of UC via the stimulation of mucus production goblet cell restitution [30], we examined mucin-1 expression with and without DSS and observed distinct colonic changes after 5 days of treatment (Supplementary Figure S2). Compared to WT and KO + VC mice, KO mice could not maintain the level of mucin-1 after being treated with DSS (Figure 4). This suggests that WT and KO + VC mice recruit a large number of NKp46(+) cells and attenuate DSS-induced inflammation and mucus depletion through the production of a large amount of IL-22, whereas KO mice lack such factors to ameliorate the colitis caused by DSS treatment.

### 2.4. Vitamin C Insufficiency and DSS Treatment Increase IL-6 Production and STAT3 and Akt Activation

**Figure 5:**
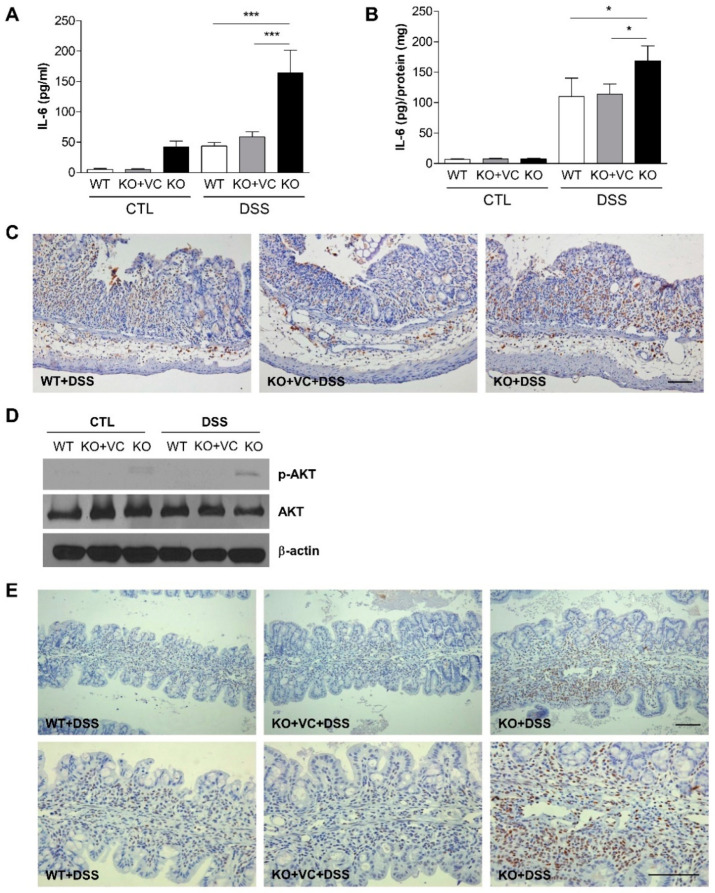
Vitamin C insufficiency and DSS treatment increase IL-6 production. Mice were treated with DSS for 7 days, and the level of IL-6 was measured in (**A**) plasma (*n* = 15–18) and (**B**) colon homogenates (*n* = 9–13) by ELISA. The final tissue IL-6 concentration was normalized to proteins in colonic homogenates. * *p* < 0.05, *** *p* < 0.001. (**C**) Infiltrated macrophages were stained with F4/80 antibodies. Scale bar, 100 μm. (**D**) Akt phosphorylation was examined in colon lysates with immunoblotting. The results are representative of three independent experiments. (**E**) Phosphorylated STAT3 in the colon was examined by immunohistochemistry. Scale bar, 100 μm. Statistical analysis was performed using the Tukey multiple comparisons test after one-way ANOVA.

Previously, we evaluated changes in various pro- and anti-inflammatory cytokines and found a notable increase in IL-6 in KO mice compared to WT or KO + VC mice with the DSS treatment. Based on this pilot result, we measured the plasma concentration of IL-6 with ELISA. Plasma IL-6 levels were high in KO mice and increased in response to the DSS treatment in the three groups of mice (Figure 5A). IL-6 levels in the colon also increased significantly due to the DSS treatment, and even more in the KO groups than in the WT and KO + VC groups (Figure 5B). The production of IL-6 in both plasma and colon tissues was highest in DSS-treated KO mice, and in addition to the IL-6 increase, a higher number of F4/80-positive macrophages accumulated in the colon of DSS-treated KO mice (Figure 5C). Furthermore, we detected that IL-6 activated three signaling pathways, Shp2-Ras-ERK, JAK1/2-STAT3, and PI3K-Akt-mTOR [31,32,33]. Thus, we found it appropriate to evaluate the activation of Akt and STAT3 in DSS-treated colonic tissue through immunohistochemistry and immunoblotting. The results demonstrated that DSS amplified the phosphorylation of Akt and STAT3, especially in KO mice (Figure 5D). STAT3 was also activated with the DSS treatment in both the intestinal epithelium and infiltration immune cells in the lamina propria, and the number of cells with phosphorylated STAT3 was higher in the KO mice than the WT and KO + VC mice (Figure 5E). These results indicate that vitamin C-insufficient KO mice produce a higher amount of colonic IL-6 in response to DSS treatment, activating the Akt and STAT3 signaling pathways.

## 3. Discussion

The present study showed that vitamin C insufficiency increased the severity of DSS-induced colitis. This effect is associated with decreased IL-22 production, leading to mucin loss in the colon epithelium as well as increased IL-6 production.

The exact mechanism related to IBD’s development is still unclear, but recent studies have shown that inflammatory reactions caused by an imbalance in the intestinal microbiome of susceptible hosts mark the beginning of IBD development [34]. ROS production, as in many immunoregulatory factors, occurs at abnormally high levels when met with IBD [9]. Additionally, irrespective of disease activity, TAC and cTAC are reduced in IBD patients [20]. Amplified oxidative stress, coupled with a reduction in antioxidant defenses, leads to an increase in damage to DNA in IBD patients [18,35,36]. Adult patients with IBD often become underweight and lack nutrients due to appetite loss, decreased intake due to abdominal pain, absorption defects, and chronic gastrointestinal defects [37,38]. Restrictive diets in the inactive stage of IBD further cases of nutritional deficiency, correlating to the depletion of vital micronutrients such as vitamin C [16,37,39,40].

After being synthesized in the kidneys or liver, vitamin C circulates into tissues. In some species, including humans, the terminal rate-limiting enzyme gluconolactone oxidase is lost. Vitamin C scavenges ROS as an antioxidant, a crucial dietary function [21,41]. Our previous reports established that an increased uptake of vitamin C by keratinocytes exposed to UVB inhibits the production of IL-8 and monocyte chemotactic protein (MCP)-1; this suggests that vitamin C acts as an anti-inflammatory agent by effectively inhibiting the inflammatory response [42]. Therefore, as an essential micronutrient with antioxidative and anti-inflammatory functions, vitamin C may be supplementary in managing IBD. Concurrent with its role as a ROS scavenger and a reducing agent, vitamin C also acts as a cofactor for the hydrolysis of lysine and proline in collagen synthesis and cross-linkage pathways. Supplementing vitamin C in some animals revealed that it is valuable: inducing collagen accumulation, promoting inflammatory rejoinders, and increasing anastomotic strength [43,44,45]. Along with clinical remission and steroid independence, mucosal healing is considered a pivotal prognostic aspect of IBD management. As vitamin C can enhance mucosal healing, it can improve outcomes for select IBD patients. A recent study examined the effect of vitamin C deficiency on the pathogenesis of UC and CD in humans via the antioxidants in vegetables containing vitamin C and miscellaneous micronutrients [46,47].

During chronic inflammation, IL-22 is induced and often protects against damaging tissues similar to IL-10, specifically exerting its effects during acute inflammation in mouse colitis models [25,30]. We found increased production of IL-22 plasma and tissue in the WT and KO+VC groups treated with DSS (Figure 3A,B). However, DSS-induced IL-22 production was significantly lower in the KO group than in the other groups. The natural killer cell expressing NKp46 on its surface plays an important role in IL-22-mediated innate intestinal immune defense [27,48]. As shown in Figure 3D, a smaller number of NKp46(+) cells infiltrated the colonic tissues of KO mice after the DSS treatment, which correlated to the decreased production of IL-22 in KO mice. Moreover, the expression of mucin-1 was decreased in the colons of DSS-treated mice (Figure 4), consistent with a report that IL-22 is dispensable for mucin production to attenuate inflammation [30]. This suggests that the impaired production of colonic IL-22 and mucin, which increases the severity of colitis, is caused by vitamin C insufficiency.

Lymphoid lineage cells, including T cells, NK cells, and ILCs—especially NKp46+ ILC3—are known as the main producers of IL-22 [49,50,51]. On this basis, we focused the experiment on the protective role of IL-22 from NKp46+ cells regulated by vitamin C on DSS-induced colitis. However, there is a report regarding the production of IL-22 from activated macrophages and mast cells [52,53], particularly regarding incidents of increased tryptase-positive mast cells in the colons of IBD patients [54,55]. In the case of mast cells in a patient’s colon, it is known that the anti-inflammatory response in the mucous membrane caused by IL-22 decreases due to the reduction in IL-22 production because of a defect in IL-33/ST2 signaling [56]. Therefore, more extensive studies about the effect of vitamin C on mast cells in the gut epithelium are required to clarify whether the IL-22-dependent anti-inflammatory effects of vitamin C on colitis are through mast cell regulation.

Both IL-22 and IL-6 activate STAT3 signaling, but epithelial STAT3 activation in DSS-induced colitis depends more on IL-22 than on IL-6 [31]. However, in this study, STAT3 phosphorylation was increased in the colons of DSS-treated KO mice, concomitant with increased IL-6 production and decreased IL-22 production. IL-22 can activate STAT3 in epithelial cells because of the limited expression of receptors. Figure 5E shows that phosphorylated STAT3 expression was higher in nonepithelial cells in the lamina propria than in the epithelium. Because DSS caused the accumulation of nonepithelial cells in the colonic tissues of KO mice, phosphorylated STAT3 expression induced by increased IL-6 may be enhanced in DSS-treated KO mice. An analysis of signal transduction pathways revealed that Akt and STAT3 were activated, whereas p38 MAPK was not activated, in DSS-treated KO mice (Figure 3E and Figure 5D,E). IL-6 activates ERK/STAT3/Akt [32], and IL-22 activates ERK/STAT3/p38 MAPK [28]. Considering the increase in IL-6 and the more minor increase in IL-22 in DSS-treated KO mice, the different activation of Akt, STAT3, and p38 MAPK is consistent with the different levels of IL-6 and IL-22. Moreover, the lower level of p38 MAPK activation might be related not only to the lower production of IL-22 in KO mice but also to decreased IL-22Rα expression in KO mice after DSS treatment (Figure 3F).

## 4. Materials and Methods

### 4.1. Animals

C57BL/6 WT mice and KO mice were maintained under specific pathogen-free conditions at the animal facility at the Seoul National University College of Medicine. Male mice (8–10 weeks old) were used for experiments. The KO group was maintained with the supplementation of vitamin C (3.3 g/L) in their drinking water to prevent death caused by vitamin C deficiency. For colitis experiments, vitamin C supplementation was discontinued for 3 weeks (KO group) or continued (KO + VC group). KO mice were not in a vitamin C-depleted state, but in a vitamin C-insufficient state, and the plasma level of vitamin C in the KO group was 1/3–1/4 of that in WT and KO + VC mice [57,58]. All experiments using animals were reviewed and approved by the Institutional Animal Care and Use Committee of Seoul National University.

### 4.2. Induction of Acute Colitis

Mice were provided 3% DSS (MP Biochemicals, Irvine, CA, USA) dissolved in drinking water for 5 or 7 days to induce acute colitis. The mice were checked daily for behavior, water and food consumption, body weight, stool consistency, and the presence of gross blood in the stool or the anus. Weight change was calculated as the percentage change in weight compared with body weight on day 0. The three groups of mice were peritoneally injected with azoxymethane (AOM, 12 mg/kg). One week after AOM injection, 2% DSS was supplemented in drinking water for 5 days. Two weeks after DSS treatment, 2% DSS has supplemented in the drinking water for 5 days once more. Lastly, after 2 weeks, 2% DSS supplementation was given to mice for 5 days. After staining with indigo carmine solution (Sigma, St. Louis, MO, USA), the number of tumors was counted.

### 4.3. Histological Evaluation of Colonic Damage

The colon samples were fixed in 4% paraformaldehyde (PFA), paraffin-embedded, and sectioned and stained with hematoxylin and eosin (H&E). A histological assessment was performed by a trained pathologist who was blind to the treatment. Histological quantification was performed in a blind method using a scoring system. Briefly, cellularity, edema, erosion (or ulceration of the mucosa), and loss of mucosal architecture, were evaluated as follows: 0, normal; 1, a slight increase in cellularity (primarily lymphocytes in the lamina propria); 2, increase in cellularity, neutrophils present, mild edema; 3, diffuse increase in cellularity, focal erosions, or ulcerations of mucosa; 4, increased cellularity, large and/or multifocal mucosal ulcerations; 5, diffuse ulceration, loss of mucosal architecture [59].

### 4.4. Enzyme-Linked Immunosorbent Assay (ELISA)

Blood was collected from the intraorbital plexuses of mice with a capillary tube and readily centrifuged at 14,000 rpm for 30 min at 4 °C. Sera of the upper layer were collected into new tubes and stored at −70 °C until use. Colonic tissues were homogenized with lysis buffer and quantified with the BCA method. The final concentrations of IL-6 (R&D system, Minneapolis, MN, USA) and IL-22 (BioLegend, San Diego, CA, USA) in the colon were normalized to the amounts of total protein in the colonic tissue lysates. ELISA was performed according to the manufacturer’s instructions.

### 4.5. Myeloperoxidase (MPO) Assay

Weighted colons were homogenized in 0.5 mL of ice-cold 0.5% hexadecyltrimethylammonium bromide (HTAB, Sigma) in 50 mM of phosphate buffer (pH 6.0). HTAB was used to negate the pseudoperoxidase activity of hemoglobin and to solubilize the membrane-bound MPO. The homogenate was centrifuged at 18,000× *g* for 20 min at 4 °C. The supernatant reacted with a mixture containing 0.167 mg/mL O-dianisidine dihydrochloride (Sigma) and 0.005% H_2_O_2_ and an amount of MPO for 10 min at room temperature. The reaction was terminated by adding 0.02% sodium azide. The absorbance was measured at 460 nm, and the amount of MPO was normalized to the weight of the colon.

### 4.6. Immunoblotting

Colonic tissues were homogenized with lysis buffer and quantified with the BCA method. Protein was mixed with 5× SDS sample buffer and loaded onto each lane of a 10% SDS-PAGE gel. Proteins were separated by electrophoresis and transferred to a nitrocellulose membrane with an electroblotting apparatus. Nonspecific sites were blocked with 5% skim milk for 1 h, and the membranes were then incubated with primary antibody against 3-nitrotyrosine (NT), p38 MAPK (Santa Cruz, Palo Alto, CA, USA), IL-22R (R&D Systems), p-Akt, Akt, p-p38 MAPK (Cell Signaling, Danvers, MA, USA), or β-actin (Sigma) at 4 °C overnight. After washing with PBS-T (0.05% Tween-20 in PBS), membranes were incubated with horseradish peroxidase-conjugated secondary antibody (Cell signaling) and detected with the ECL detection kit (Amersham, Piscataway, NJ, USA).

### 4.7. Immunohistochemistry

Colon tissues were freshly isolated and fixed in 4% PFA at 4 °C. Paraffin-embedded tissues were sectioned at a thickness of 4 μm. After deparaffinization and hydration, the antigen epitope was retrieved by heating it in 0.1 M citrate buffer (pH 6.0) in a microwave. After blocking endogenous peroxidase with H_2_O_2_ and inhibiting nonspecific signals with 5% goat serum, sections were incubated with primary antibodies against p-STAT3 (Santa Cruz), Gr1 (MACS, Bergisch Gladbach, Germany), NKp46 (BD Bioscience, San Jose, CA, USA), mucin-1 (Abcam, Cambridge, MA, USA), or IL-22 (R&D Systems) at 4 °C overnight in a humidified chamber. Then, sections were incubated with the corresponding biotinylated secondary antibody (Vector Laboratories, Burlingame, CA, USA) for 1 h at room temperature. ABC solution (Vector Laboratories) was loaded on sections for 30 min, and a DAB kit (Vector Laboratories) was used for chromogenic detection. Subsequent to dehydration and clearing, the sections were mounted with DPX mounting medium (Fluka, St. Louis, MO, USA) and observed under a light microscope (Olympus, Center Valley, PA, USA).

### 4.8. Statics

Data from independent experiments are expressed as the mean ± SEM of each group. For comparisons of three or more groups, data were analyzed using the Tukey multiple comparisons test after one-way ANOVA. *p* < 0.05 was considered statistically significant. Statistical tests were performed using GraphPad InStat (GraphPad Software, San Diego, CA, USA).

## 5. Conclusions

Vitamin C insufficiency increased inflammatory cell infiltration and oxidative stress. Vitamin C insufficiency resulted in decreased IL-22 and mucin production and increased the production of IL-6 in the DSS-induced colons of vitamin C-insufficient KO mice, which seems to cause severe colitis. These results suggest that vitamin C is a potential treatment for IBD patients due to its antioxidant and anti-inflammatory effects.

## Data Availability

Not applicable.

## Supplementary Materials

The following supporting information can be downloaded at: https://www.mdpi.com/article/10.3390/ijms231810612/s1.
