# Supplementary material for: Preventive Effect of Vitamin C on Dextran Sulfate Sodium (DSS)-Induced Colitis via the Regulation of IL-22 and IL-6 Production in Gulo(−/−) Mice
Source: Int J Mol Sci. 2022 Sep 13;23(18):10612. doi: 10.3390/ijms231810612 (PMC9505994; doi:10.3390/ijms231810612)
Supplement: Supplementary file 1 [file ijms-23-10612-s001.zip › ijms-1690109-supplementary.pdf]

S1

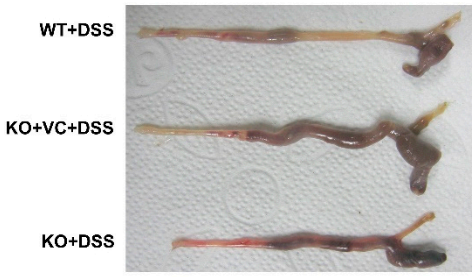

Figure S1. The length of the colon from the anus to the ileocecal valve in DSS-treated mice.

S2

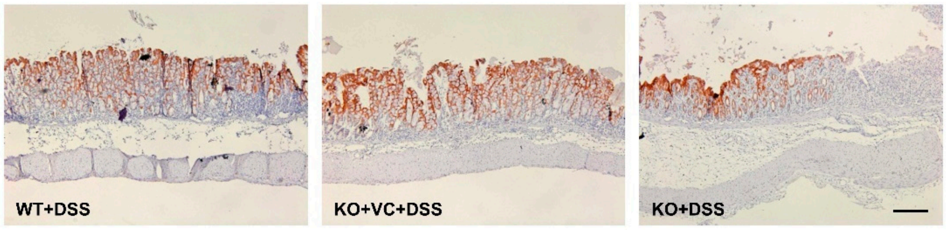

Figure S2. The distribution of mucin-1 expression after DSS treatment for 5 days.
